# Dual systemic allergic rhinitis and local allergic rhinitis

**DOI:** 10.1186/1939-4551-8-S1-A262

**Published:** 2015-04-08

**Authors:** Miguel Blanca, Paloma Campo, Carmen Rondon, Esther Barrionuevo Sanchez, Natalia Blanca-López, Veronique Godineau, Maria Auxiliadora Guerrero, Maria Jose Torres

**Affiliations:** 1 Allergy Unit, Regional University Hospital of Málaga, Spain; 2 Allergy Service, Hospital Infanta Leonor, Spain; 3 Research Laboratory, Regional University Hospital of Málaga, Spain

## Rationale

The aim of this study was investigated if seasonal systemic allergic rhinitis (SAR) induced by pollens and local allergic rhinitis (LAR) induced by perennial allergens may coexist in the same patient.

## Methods

Twenty-nine patients with seasonal SAR (positive SPT to grass and/or olive pollen) with well-defined symptoms during pollen season in addition to nasal symptoms throughout the year were included. Clinical questionnaires, skin tests, serum specific IgE, nasal levels of tryptase and eosinophil cationic protein, and nasal allergen provocation test (NAPT) with grass, olive, *D. pteronyssinus* (DP), and *Alternaria alternata* (AA) were performed.

## Results

The coexistence of dual SAR-LAR was confirmed in 23 patients by NAPT (82.1%) with positive response to AA in 9 patients (22%), to DP in 19 (46.3%), and to both AA and DP in 13 (31.7%). No discordance between SPT and NAPT results was obtained. The SPT and NAPT with seasonal pollens were positive to grass in 11 patients (26.8%), to olive in 20 (48.8%), and to both grass and olive in 11 (26.8%). No significant differences between seasonal and perennial allergens threshold concentrations were observed. The 56.5% of patients reported a seasonal onset of nasal symptom followed by perennial symptoms in the next years. The 30.4% a perennial onset of symptoms with a clear spring worsening, and the 13% could not remember the onset of the disease.

## Conclusions

These results demonstrate the coexistence of seasonal SAR and perennial LAR in patients who developing symptoms throughout the year and have negative SPT to perennial allergens should be explored.

